# Cytenamide–formic acid (1/1)

**DOI:** 10.1107/S1600536808019181

**Published:** 2008-07-05

**Authors:** Andrea Johnston, Alastair J. Florence, Gary J. Miller, Alan R. Kennedy, Colin T. Bedford

**Affiliations:** a Solid-State Research Group, Strathclyde Institute of Pharmacy and Biomedical Sciences, The John Arbuthnott Building, University of Strathclyde, 27 Taylor Street, Glasgow G4 0NR, Scotland; b WestCHEM, Department of Pure & Applied Chemistry, University of Strathclyde, 295 Cathedral Street, Glasgow G1 1XL, Scotland; c University College London, Department of Chemistry, 20 Gordon Street, London WC1H 0AJ, England

## Abstract

In the crystal structure of the title compound [systematic name: 5*H*-dibenzo[*a*,*d*]cyclo­hepta­triene-5-carboxamide–meth­anoic acid (1/1)], C_16_H_13_NO·CH_2_O_2_, the cytenamide and solvent mol­ecules form a hydrogen-bonded *R*_2_^2^(8) dimer motif, which is further connected to form a centrosymmetric double-motif arrangement. The asymmetric unit contains two formula units.

## Related literature

For details on experimental methods used to obtain this form, see: Davis *et al.* (1964 ▶); Florence *et al.* (2003 ▶); Florence, Johnston, Fernandes *et al. *(2006 ▶). For related literature on cytenamide, see: Florence, Bedford *et al.* (2008 ▶). For cyten­amide analogues, see: Cyr *et al.* (1987 ▶); Fleischman *et al.* (2003 ▶); Florence, Johnston, Price *et al.* (2006 ▶); Florence, Leech *et al.* (2007 ▶); Bandoli *et al.* (1992 ▶); Harrison *et al.* (2006 ▶); Leech *et al.* (2006 ▶); Florence, Shankland *et al.* (2008 ▶). For graph-set motifs, see: Etter (1990 ▶).
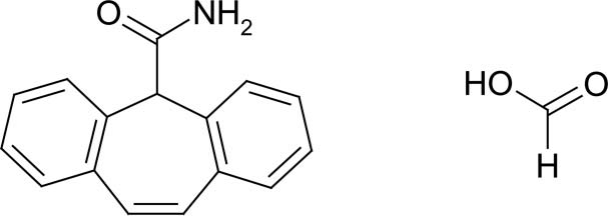


## Experimental

### 

#### Crystal data

C_16_H_13_NO·CH_2_O_2_
*M* *_r_* = 281.3
Monoclinic, 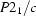

*a* = 11.5351 (13) Å
*b* = 13.9095 (15) Å
*c* = 17.6904 (19) Å
β = 95.846 (5)°
*V* = 2823.6 (5) Å^3^
*Z* = 8
Mo *K*α radiation
μ = 0.09 mm^−1^
*T* = 123 (2) K
0.25 × 0.15 × 0.05 mm

#### Data collection

Bruker APEXII CCD diffractometer
Absorption correction: multi-scan (*SADABS*; Sheldrick, 2002 ▶) *T*_min_ = 0.978, *T*_max_ = 0.996
55762 measured reflections
12996 independent reflections
9356 reflections with *I* > 2/s(*I*)
*R*_int_ = 0.025

#### Refinement

*R*[*F* ^2^ > 2σ(*F* ^2^)] = 0.047
*wR*(*F* ^2^) = 0.142
*S* = 1.02
12996 reflections
411 parameters
H atoms treated by a mixture of independent and constrained refinement
Δρ_max_ = 0.58 e Å^−3^
Δρ_min_ = −0.23 e Å^−3^

### 

Data collection: *APEX2* (Bruker, 2007 ▶); cell refinement: *APEX2*; data reduction: *SAINT* (Bruker, 2007 ▶); program(s) used to solve structure: *SHELXS97* (Sheldrick, 2008 ▶); program(s) used to refine structure: *SHELXL97* (Sheldrick, 2008 ▶); molecular graphics: *ORTEP-3* (Farrugia, 1997 ▶) and *Mercury* (Macrae *et al.*, 2006 ▶); software used to prepare material for publication: *PLATON* (Spek, 2003 ▶).

## Supplementary Material

Crystal structure: contains datablocks global, I. DOI: 10.1107/S1600536808019181/rn2044sup1.cif

Structure factors: contains datablocks I. DOI: 10.1107/S1600536808019181/rn2044Isup2.hkl

Additional supplementary materials:  crystallographic information; 3D view; checkCIF report

## Figures and Tables

**Table 1:** Hydrogen-bond geometry (Å, °)

*D*—H⋯*A*	*D*—H	H⋯*A*	*D*⋯*A*	*D*—H⋯*A*
N1—H1*N*⋯O4	0.884 (15)	2.035 (15)	2.9096 (12)	170.2 (13)
O3—H1*O*⋯O1	0.927 (18)	1.679 (19)	2.5971 (12)	169.9 (18)
O6—H2*O*⋯O2	0.91 (2)	1.66 (2)	2.5517 (12)	168.3 (19)
N2—H3*N*⋯O5	0.895 (15)	2.103 (15)	2.9645 (12)	161.2 (14)
N2—H4*N*⋯O4	0.843 (16)	2.237 (15)	2.9129 (12)	137.3 (13)
N1—H2*N*⋯O5	0.866 (16)	2.151 (16)	2.9088 (12)	145.9 (13)

**Fig. 1.**
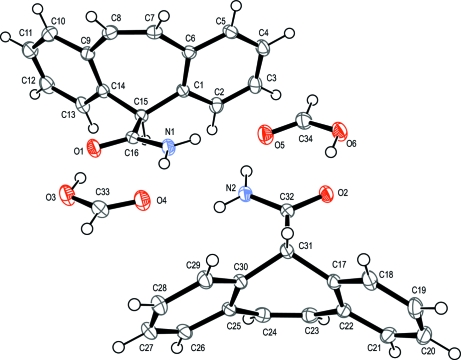
The molecular structure and atomic labelling of CYT formic acid, showing 50% probability displacement ellipsoids.

**Fig. 2.**
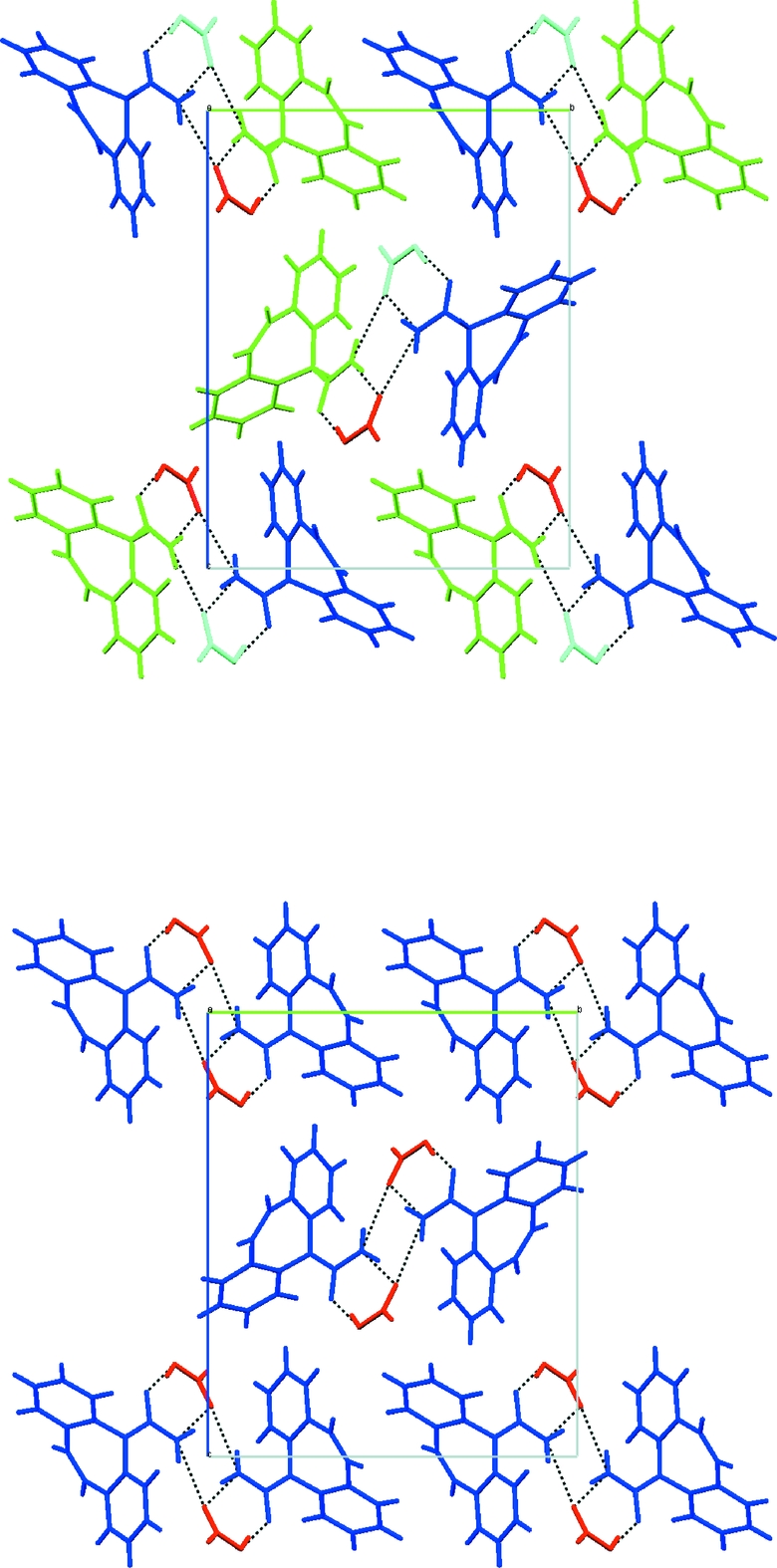
The crystal packing in CYT-formic acid (top) and CBZ-formic acid (bottom), viewed down the a-axis. Molecules are coloured according to symmetry equivalence.

## supplementary crystallographic information

### Comment

Cytenamide (CYT) is an analogue of carbamazepine (CBZ), a dibenzazepine drug used to control seizures (Cyr *et al.*, 1987). CYT-formic acid solvate was produced during an automated parallel crystallization study (Florence *et al.*, 2006) of CYT as part of a wider investigation that couples automated parallel crystallization with crystal structure prediction methodology to investigate the basic science underlying the solid-state diversity of CBZ (Florence, Johnston, Price *et al.*, 2006; Florence, Leech *et al.*, 2007) and its closely related analogues: CYT (Florence, Bedford *et al.*, 2008), 10,11-dihydrocarbamazepine (DHC) (Bandoli *et al.*, 1992; Harrison *et al.*, 2006; Leech *et al.*, 2006) and cyheptamide (Florence, Shankland *et al.*, 2008). The sample was identified as a new form using multi-sample foil transmission X-ray powder diffraction analysis (Florence *et al.*, 2003). Subsequent manual recrystallization from a saturated formic acid solution by slow evaporation at 278 K yielded a sample suitable for single-crystal X-ray diffraction (Fig. 1).

The molecules crystallize in the space group *P*2_1_/c with two CYT and two solvent molecules in the asymmetric unit. Both CYT molecules form an *R*_2_^2^(8) (Etter, 1990) dimer motif with adjacent solvent molecules *via* contacts 1 - 4 (Table 1). In addition, two N—H···O contacts (5 and 6) join adjacent dimers to form a *R*_4_^2^(8) centrosymmetric double motif (Fig. 2).

This packing arrangement is similar to that in CBZ-formic acid solvate which, in contrast, crystallizes with *Z*' = 1 in the monoclinic space group *P*2_1_/c (Fig. 2). The main difference being a doubling of the *a* axis in CYT-formic acid solvate (*Z*' = 2) (Fleischman *et al.*, 2003)

### Experimental

A sample of cytenamide was synthesized according to a modification of the published method (Davis *et al.*, 1964). A single-crystal sample of the title compound was recrystallized from a saturated formic acid solution by isothermal solvent evaporation at 278 *^o^*K.

### Crystal data

**Table:**

C_16_H_13_NO·CH_2_O_2_	*F*_000_ = 1184
*M**_r_* = 281.3	*D*_x_ = 1.323 Mg m^−^^3^
Monoclinic, *P*2_1_/*c*	Mo *K*α radiation λ = 0.71073 Å
Hall symbol: -P 2ybc	Cell parameters from 9893 reflections
*a* = 11.5351 (13) Å	θ = 2.5–35.6º
*b* = 13.9095 (15) Å	µ = 0.09 mm^−^^1^
*c* = 17.6904 (19) Å	*T* = 123 (2) K
β = 95.846 (5)º	Block, colourless
*V* = 2823.6 (5) Å^3^	0.25 × 0.15 × 0.05 mm
*Z* = 8	

### Data collection

**Table:**

Bruker APEXII CCD diffractometer	12996 independent reflections
Radiation source: fine-focus sealed tube	9356 reflections with *I* > 2/s(*I*)
Monochromator: graphite	*R*_int_ = 0.025
*T* = 123(2) K	θ_max_ = 35.7º
φ and ω scans	θ_min_ = 2.3º
Absorption correction: Multi-scan(SADABS; Sheldrick, 2002)	*h* = −18→18
*T*_min_ = 0.978, *T*_max_ = 0.996	*k* = −18→22
55762 measured reflections	*l* = −28→28

### Refinement

**Table:**

Refinement on *F*^2^	Secondary atom site location: difference Fourier map
Least-squares matrix: full	Hydrogen site location: inferred from neighbouring sites
*R*[*F*^2^ > 2σ(*F*^2^)] = 0.047	H atoms treated by a mixture of independent and constrained refinement
*wR*(*F*^2^) = 0.142	*w* = 1/[σ^2^(*F*_o_^2^) + (0.0728*P*)^2^ + 0.6562*P*] where *P* = (*F*_o_^2^ + 2*F*_c_^2^)/3
*S* = 1.02	(Δ/σ)_max_ = 0.002
12996 reflections	Δρ_max_ = 0.58 e Å^−^^3^
411 parameters	Δρ_min_ = −0.23 e Å^−^^3^
Primary atom site location: structure-invariant direct methods	Extinction correction: none

### Special details

**Table:**

Geometry. All e.s.d.'s (except the e.s.d. in the dihedral angle between two l.s. planes) are estimated using the full covariance matrix. The cell e.s.d.'s are taken into account individually in the estimation of e.s.d.'s in distances, angles and torsion angles; correlations between e.s.d.'s in cell parameters are only used when they are defined by crystal symmetry. An approximate (isotropic) treatment of cell e.s.d.'s is used for estimating e.s.d.'s involving l.s. planes.
Refinement. Refinement of *F*^2^ against ALL reflections. The weighted *R*-factor *wR* and goodness of fit *S* are based on *F*^2^, conventional *R*-factors *R* are based on *F*, with *F* set to zero for negative *F*^2^. The threshold expression of *F*^2^ > σ(*F*^2^) is used only for calculating *R*-factors(gt) *etc*. and is not relevant to the choice of reflections for refinement. *R*-factors based on *F*^2^ are statistically about twice as large as those based on *F*, and *R*- factors based on ALL data will be even larger.

### Fractional atomic coordinates and isotropic or equivalent isotropic displacement parameters (Å^2^)

**Table:**

	*x*	*y*	*z*	*U*_iso_*/*U*_eq_	
O1	0.36614 (7)	0.30926 (5)	0.65575 (4)	0.02658 (15)	
O2	0.10330 (7)	0.67015 (5)	0.37463 (4)	0.02342 (14)	
O3	0.19631 (8)	0.37994 (6)	0.72402 (4)	0.02951 (16)	
O4	0.14455 (7)	0.47636 (6)	0.62510 (4)	0.02896 (16)	
O5	0.30232 (7)	0.49021 (6)	0.40062 (4)	0.03007 (17)	
O6	0.23678 (8)	0.57335 (6)	0.29680 (4)	0.03242 (18)	
N1	0.33490 (8)	0.39736 (6)	0.54859 (5)	0.02341 (16)	
N2	0.11769 (8)	0.58059 (5)	0.48157 (5)	0.02066 (15)	
C1	0.50028 (8)	0.29386 (6)	0.47664 (5)	0.01748 (14)	
C2	0.57461 (8)	0.35519 (7)	0.44219 (5)	0.02202 (17)	
H2	0.6303	0.3921	0.4731	0.026*	
C3	0.56919 (9)	0.36362 (8)	0.36373 (6)	0.02555 (19)	
H3	0.6216	0.4050	0.3413	0.031*	
C4	0.48681 (10)	0.31127 (7)	0.31820 (6)	0.02523 (19)	
H4	0.4826	0.3166	0.2645	0.030*	
C5	0.41066 (9)	0.25107 (7)	0.35153 (5)	0.02272 (17)	
H5	0.3528	0.2170	0.3201	0.027*	
C6	0.41737 (8)	0.23938 (6)	0.43097 (5)	0.01853 (15)	
C7	0.33783 (8)	0.17138 (6)	0.46164 (6)	0.02090 (16)	
H7	0.2639	0.1641	0.4332	0.025*	
C8	0.35587 (9)	0.11767 (6)	0.52507 (6)	0.02140 (16)	
H8	0.2934	0.0769	0.5355	0.026*	
C9	0.45948 (8)	0.11419 (6)	0.57986 (5)	0.02008 (16)	
C10	0.48622 (11)	0.02677 (7)	0.61783 (6)	0.0281 (2)	
H10	0.4344	−0.0261	0.6094	0.034*	
C11	0.58653 (11)	0.01640 (7)	0.66714 (6)	0.0307 (2)	
H11	0.6032	−0.0431	0.6923	0.037*	
C12	0.66267 (10)	0.09340 (8)	0.67957 (6)	0.0282 (2)	
H12	0.7329	0.0861	0.7120	0.034*	
C13	0.63592 (9)	0.18121 (7)	0.64440 (5)	0.02332 (17)	
H13	0.6877	0.2340	0.6537	0.028*	
C14	0.53432 (8)	0.19277 (6)	0.59577 (5)	0.01848 (15)	
C15	0.50395 (8)	0.29080 (6)	0.56232 (5)	0.01808 (15)	
H15	0.5692	0.3343	0.5819	0.022*	
C16	0.39403 (8)	0.33227 (6)	0.59203 (5)	0.01896 (15)	
C17	−0.00637 (8)	0.82307 (6)	0.44107 (5)	0.01786 (15)	
C18	−0.10409 (9)	0.85561 (7)	0.39547 (5)	0.02321 (17)	
H18	−0.1708	0.8155	0.3872	0.028*	
C19	−0.10533 (10)	0.94622 (8)	0.36176 (6)	0.0293 (2)	
H19	−0.1734	0.9683	0.3321	0.035*	
C20	−0.00710 (11)	1.00399 (7)	0.37167 (6)	0.0306 (2)	
H20	−0.0070	1.0654	0.3481	0.037*	
C21	0.09067 (10)	0.97183 (7)	0.41602 (6)	0.0263 (2)	
H21	0.1585	1.0110	0.4216	0.032*	
C22	0.09204 (8)	0.88216 (6)	0.45311 (5)	0.01954 (16)	
C23	0.19633 (9)	0.85676 (7)	0.50299 (6)	0.02165 (16)	
H23	0.2679	0.8812	0.4887	0.026*	
C24	0.20378 (8)	0.80303 (6)	0.56672 (5)	0.02076 (16)	
H24	0.2799	0.7946	0.5918	0.025*	
C25	0.10967 (8)	0.75612 (6)	0.60205 (5)	0.01819 (15)	
C26	0.12267 (9)	0.74452 (7)	0.68148 (5)	0.02342 (18)	
H26	0.1936	0.7635	0.7095	0.028*	
C27	0.03445 (11)	0.70599 (7)	0.71980 (5)	0.0276 (2)	
H27	0.0446	0.6996	0.7735	0.033*	
C28	−0.06874 (10)	0.67688 (8)	0.67918 (6)	0.02705 (19)	
H28	−0.1303	0.6518	0.7051	0.032*	
C29	−0.08193 (9)	0.68443 (7)	0.60040 (5)	0.02195 (17)	
H29	−0.1521	0.6629	0.5729	0.026*	
C30	0.00612 (8)	0.72303 (6)	0.56107 (5)	0.01725 (14)	
C31	−0.00718 (8)	0.72319 (6)	0.47516 (5)	0.01655 (14)	
H31	−0.0865	0.6964	0.4595	0.020*	
C32	0.07859 (8)	0.65615 (6)	0.44077 (5)	0.01707 (14)	
C33	0.13195 (9)	0.44576 (7)	0.68783 (6)	0.02455 (18)	
C34	0.30291 (11)	0.50955 (7)	0.33411 (6)	0.0285 (2)	
H1N	0.2766 (13)	0.4268 (10)	0.5677 (8)	0.029 (3)*	
H2N	0.3531 (13)	0.4124 (11)	0.5038 (9)	0.034 (4)*	
H3N	0.1661 (13)	0.5407 (11)	0.4605 (8)	0.029 (3)*	
H4N	0.1007 (13)	0.5752 (10)	0.5266 (9)	0.029 (4)*	
H34	0.3583 (14)	0.4788 (12)	0.3007 (10)	0.043 (4)*	
H33	0.0717 (13)	0.4695 (10)	0.7176 (8)	0.029 (3)*	
H1O	0.2537 (16)	0.3588 (14)	0.6949 (10)	0.053 (5)*	
H2O	0.1899 (17)	0.6011 (14)	0.3288 (11)	0.057 (5)*	

### Atomic displacement parameters (Å^2^)

**Table:**

	*U*^11^	*U*^22^	*U*^33^	*U*^12^	*U*^13^	*U*^23^
O1	0.0355 (4)	0.0284 (3)	0.0168 (3)	0.0098 (3)	0.0073 (3)	0.0041 (2)
O2	0.0336 (4)	0.0227 (3)	0.0146 (3)	0.0073 (3)	0.0055 (3)	0.0018 (2)
O3	0.0336 (4)	0.0363 (4)	0.0190 (3)	0.0070 (3)	0.0042 (3)	0.0048 (3)
O4	0.0346 (4)	0.0314 (3)	0.0216 (3)	0.0098 (3)	0.0064 (3)	0.0046 (3)
O5	0.0367 (4)	0.0318 (4)	0.0218 (3)	0.0112 (3)	0.0033 (3)	0.0044 (3)
O6	0.0484 (5)	0.0313 (4)	0.0181 (3)	0.0163 (3)	0.0061 (3)	0.0017 (3)
N1	0.0319 (4)	0.0219 (3)	0.0171 (3)	0.0081 (3)	0.0055 (3)	0.0027 (3)
N2	0.0279 (4)	0.0188 (3)	0.0154 (3)	0.0048 (3)	0.0030 (3)	0.0018 (2)
C1	0.0186 (4)	0.0186 (3)	0.0152 (3)	0.0005 (3)	0.0015 (3)	0.0000 (3)
C2	0.0198 (4)	0.0244 (4)	0.0219 (4)	−0.0018 (3)	0.0023 (3)	0.0028 (3)
C3	0.0260 (5)	0.0298 (4)	0.0220 (4)	0.0015 (4)	0.0080 (4)	0.0050 (3)
C4	0.0325 (5)	0.0270 (4)	0.0169 (4)	0.0063 (4)	0.0064 (3)	0.0006 (3)
C5	0.0285 (5)	0.0217 (4)	0.0176 (4)	0.0025 (3)	0.0005 (3)	−0.0035 (3)
C6	0.0198 (4)	0.0181 (3)	0.0177 (4)	0.0011 (3)	0.0022 (3)	−0.0019 (3)
C7	0.0201 (4)	0.0200 (3)	0.0222 (4)	−0.0022 (3)	0.0005 (3)	−0.0029 (3)
C8	0.0218 (4)	0.0189 (3)	0.0239 (4)	−0.0020 (3)	0.0042 (3)	−0.0019 (3)
C9	0.0233 (4)	0.0186 (3)	0.0188 (4)	0.0018 (3)	0.0045 (3)	−0.0006 (3)
C10	0.0395 (6)	0.0184 (4)	0.0266 (5)	0.0036 (4)	0.0037 (4)	0.0008 (3)
C11	0.0432 (6)	0.0241 (4)	0.0245 (5)	0.0135 (4)	0.0020 (4)	0.0023 (3)
C12	0.0300 (5)	0.0342 (5)	0.0204 (4)	0.0128 (4)	0.0019 (4)	0.0017 (4)
C13	0.0219 (4)	0.0305 (4)	0.0175 (4)	0.0035 (3)	0.0016 (3)	0.0014 (3)
C14	0.0195 (4)	0.0208 (3)	0.0155 (3)	0.0020 (3)	0.0038 (3)	0.0000 (3)
C15	0.0198 (4)	0.0187 (3)	0.0156 (3)	−0.0019 (3)	0.0007 (3)	−0.0004 (3)
C16	0.0250 (4)	0.0168 (3)	0.0149 (3)	0.0008 (3)	0.0012 (3)	−0.0018 (3)
C17	0.0212 (4)	0.0191 (3)	0.0137 (3)	0.0049 (3)	0.0042 (3)	0.0008 (3)
C18	0.0239 (4)	0.0300 (4)	0.0160 (4)	0.0089 (3)	0.0034 (3)	0.0027 (3)
C19	0.0377 (6)	0.0325 (5)	0.0186 (4)	0.0182 (4)	0.0071 (4)	0.0063 (3)
C20	0.0505 (7)	0.0216 (4)	0.0219 (4)	0.0135 (4)	0.0140 (4)	0.0056 (3)
C21	0.0401 (6)	0.0175 (3)	0.0232 (4)	0.0015 (3)	0.0123 (4)	0.0011 (3)
C22	0.0254 (4)	0.0172 (3)	0.0170 (4)	0.0030 (3)	0.0069 (3)	0.0000 (3)
C23	0.0218 (4)	0.0209 (3)	0.0228 (4)	−0.0014 (3)	0.0053 (3)	−0.0028 (3)
C24	0.0191 (4)	0.0214 (3)	0.0214 (4)	0.0011 (3)	0.0004 (3)	−0.0034 (3)
C25	0.0224 (4)	0.0174 (3)	0.0144 (3)	0.0031 (3)	0.0004 (3)	−0.0015 (3)
C26	0.0310 (5)	0.0224 (4)	0.0160 (4)	0.0028 (3)	−0.0021 (3)	−0.0026 (3)
C27	0.0418 (6)	0.0274 (4)	0.0138 (4)	0.0026 (4)	0.0045 (4)	−0.0012 (3)
C28	0.0345 (5)	0.0297 (4)	0.0182 (4)	−0.0001 (4)	0.0091 (4)	0.0016 (3)
C29	0.0244 (4)	0.0245 (4)	0.0175 (4)	−0.0001 (3)	0.0046 (3)	0.0013 (3)
C30	0.0208 (4)	0.0172 (3)	0.0139 (3)	0.0020 (3)	0.0022 (3)	0.0001 (3)
C31	0.0180 (4)	0.0187 (3)	0.0129 (3)	0.0008 (3)	0.0013 (3)	0.0005 (3)
C32	0.0200 (4)	0.0168 (3)	0.0141 (3)	0.0006 (3)	0.0002 (3)	−0.0005 (3)
C33	0.0256 (5)	0.0288 (4)	0.0190 (4)	0.0013 (3)	0.0014 (3)	−0.0025 (3)
C34	0.0377 (6)	0.0260 (4)	0.0221 (4)	0.0095 (4)	0.0044 (4)	−0.0006 (3)

### Geometric parameters (Å, °)

**Table:**

O1—C16	1.2451 (11)	C12—H12	0.9500
O2—C32	1.2473 (11)	C13—C14	1.3907 (13)
O3—C33	1.3047 (13)	C13—H13	0.9500
O3—H1O	0.926 (19)	C14—C15	1.5132 (12)
O4—C33	1.2111 (13)	C15—C16	1.5338 (13)
O5—C34	1.2076 (13)	C15—H15	1.0000
O6—C34	1.3048 (13)	C17—C18	1.3932 (13)
O6—H2O	0.91 (2)	C17—C22	1.4000 (13)
N1—C16	1.3300 (12)	C17—C31	1.5149 (12)
N1—H1N	0.884 (15)	C18—C19	1.3937 (14)
N1—H2N	0.866 (16)	C18—H18	0.9500
N2—C32	1.3277 (11)	C19—C20	1.3855 (19)
N2—H3N	0.895 (15)	C19—H19	0.9500
N2—H4N	0.843 (15)	C20—C21	1.3811 (16)
C1—C2	1.3931 (13)	C20—H20	0.9500
C1—C6	1.4086 (12)	C21—C22	1.4087 (13)
C1—C15	1.5126 (12)	C21—H21	0.9500
C2—C3	1.3879 (14)	C22—C23	1.4611 (14)
C2—H2	0.9500	C23—C24	1.3480 (14)
C3—C4	1.3878 (15)	C23—H23	0.9500
C3—H3	0.9500	C24—C25	1.4604 (13)
C4—C5	1.3877 (15)	C24—H24	0.9500
C4—H4	0.9500	C25—C26	1.4072 (13)
C5—C6	1.4091 (13)	C25—C30	1.4099 (13)
C5—H5	0.9500	C26—C27	1.3867 (16)
C6—C7	1.4601 (13)	C26—H26	0.9500
C7—C8	1.3465 (14)	C27—C28	1.3867 (16)
C7—H7	0.9500	C27—H27	0.9500
C8—C9	1.4608 (14)	C28—C29	1.3904 (14)
C8—H8	0.9500	C28—H28	0.9500
C9—C14	1.4034 (13)	C29—C30	1.3956 (13)
C9—C10	1.4082 (13)	C29—H29	0.9500
C10—C11	1.3838 (16)	C30—C31	1.5119 (12)
C10—H10	0.9500	C31—C32	1.5300 (12)
C11—C12	1.3883 (17)	C31—H31	1.0000
C11—H11	0.9500	C33—H33	0.972 (15)
C12—C13	1.3911 (14)	C34—H34	1.008 (17)
			
C33—O3—H1O	110.6 (11)	N1—C16—C15	116.82 (8)
C34—O6—H2O	109.2 (12)	C18—C17—C22	119.54 (8)
C16—N1—H1N	117.3 (9)	C18—C17—C31	119.41 (8)
C16—N1—H2N	122.4 (10)	C22—C17—C31	121.04 (8)
H1N—N1—H2N	120.3 (14)	C17—C18—C19	120.93 (10)
C32—N2—H3N	117.1 (9)	C17—C18—H18	119.5
C32—N2—H4N	119.1 (10)	C19—C18—H18	119.5
H3N—N2—H4N	123.6 (13)	C20—C19—C18	119.85 (10)
C2—C1—C6	119.38 (8)	C20—C19—H19	120.1
C2—C1—C15	120.00 (8)	C18—C19—H19	120.1
C6—C1—C15	120.50 (8)	C21—C20—C19	119.61 (9)
C3—C2—C1	121.48 (9)	C21—C20—H20	120.2
C3—C2—H2	119.3	C19—C20—H20	120.2
C1—C2—H2	119.3	C20—C21—C22	121.40 (10)
C4—C3—C2	119.63 (9)	C20—C21—H21	119.3
C4—C3—H3	120.2	C22—C21—H21	119.3
C2—C3—H3	120.2	C17—C22—C21	118.59 (9)
C5—C4—C3	119.71 (9)	C17—C22—C23	123.70 (8)
C5—C4—H4	120.1	C21—C22—C23	117.70 (9)
C3—C4—H4	120.1	C24—C23—C22	128.14 (9)
C4—C5—C6	121.37 (9)	C24—C23—H23	115.9
C4—C5—H5	119.3	C22—C23—H23	115.9
C6—C5—H5	119.3	C23—C24—C25	128.22 (9)
C1—C6—C5	118.36 (8)	C23—C24—H24	115.9
C1—C6—C7	123.44 (8)	C25—C24—H24	115.9
C5—C6—C7	118.20 (8)	C26—C25—C30	118.33 (9)
C8—C7—C6	128.27 (9)	C26—C25—C24	118.05 (8)
C8—C7—H7	115.9	C30—C25—C24	123.61 (8)
C6—C7—H7	115.9	C27—C26—C25	121.58 (9)
C7—C8—C9	128.16 (9)	C27—C26—H26	119.2
C7—C8—H8	115.9	C25—C26—H26	119.2
C9—C8—H8	115.9	C28—C27—C26	119.57 (9)
C14—C9—C10	118.46 (9)	C28—C27—H27	120.2
C14—C9—C8	123.52 (8)	C26—C27—H27	120.2
C10—C9—C8	118.02 (9)	C27—C28—C29	119.86 (10)
C11—C10—C9	121.28 (10)	C27—C28—H28	120.1
C11—C10—H10	119.4	C29—C28—H28	120.1
C9—C10—H10	119.4	C28—C29—C30	121.19 (9)
C10—C11—C12	119.63 (9)	C28—C29—H29	119.4
C10—C11—H11	120.2	C30—C29—H29	119.4
C12—C11—H11	120.2	C29—C30—C25	119.38 (8)
C11—C12—C13	119.91 (10)	C29—C30—C31	119.82 (8)
C11—C12—H12	120.0	C25—C30—C31	120.68 (8)
C13—C12—H12	120.0	C30—C31—C17	113.42 (7)
C14—C13—C12	120.85 (10)	C30—C31—C32	113.29 (7)
C14—C13—H13	119.6	C17—C31—C32	111.73 (7)
C12—C13—H13	119.6	C30—C31—H31	105.9
C13—C14—C9	119.76 (8)	C17—C31—H31	105.9
C13—C14—C15	119.54 (8)	C32—C31—H31	105.9
C9—C14—C15	120.68 (8)	O2—C32—N2	122.41 (8)
C1—C15—C14	113.51 (7)	O2—C32—C31	119.78 (7)
C1—C15—C16	113.15 (7)	N2—C32—C31	117.64 (8)
C14—C15—C16	111.80 (7)	O4—C33—O3	125.51 (10)
C1—C15—H15	105.9	O4—C33—H33	122.5 (9)
C14—C15—H15	105.9	O3—C33—H33	112.0 (9)
C16—C15—H15	105.9	O5—C34—O6	125.69 (10)
O1—C16—N1	122.24 (9)	O5—C34—H34	122.9 (10)
O1—C16—C15	120.82 (8)	O6—C34—H34	111.4 (9)
			
C6—C1—C2—C3	−0.25 (14)	C22—C17—C18—C19	−0.09 (13)
C15—C1—C2—C3	−176.46 (9)	C31—C17—C18—C19	−178.49 (8)
C1—C2—C3—C4	1.11 (15)	C17—C18—C19—C20	1.91 (15)
C2—C3—C4—C5	0.03 (15)	C18—C19—C20—C21	−1.09 (15)
C3—C4—C5—C6	−2.05 (15)	C19—C20—C21—C22	−1.53 (15)
C2—C1—C6—C5	−1.70 (13)	C18—C17—C22—C21	−2.46 (13)
C15—C1—C6—C5	174.50 (8)	C31—C17—C22—C21	175.92 (8)
C2—C1—C6—C7	178.35 (9)	C18—C17—C22—C23	176.71 (8)
C15—C1—C6—C7	−5.46 (13)	C31—C17—C22—C23	−4.92 (13)
C4—C5—C6—C1	2.87 (14)	C20—C21—C22—C17	3.31 (14)
C4—C5—C6—C7	−177.17 (9)	C20—C21—C22—C23	−175.91 (9)
C1—C6—C7—C8	−31.43 (15)	C17—C22—C23—C24	−31.25 (15)
C5—C6—C7—C8	148.62 (10)	C21—C22—C23—C24	147.93 (10)
C6—C7—C8—C9	0.51 (16)	C22—C23—C24—C25	0.29 (16)
C7—C8—C9—C14	30.22 (15)	C23—C24—C25—C26	−149.76 (10)
C7—C8—C9—C10	−148.87 (10)	C23—C24—C25—C30	29.39 (14)
C14—C9—C10—C11	−2.97 (15)	C30—C25—C26—C27	−3.13 (13)
C8—C9—C10—C11	176.17 (10)	C24—C25—C26—C27	176.06 (9)
C9—C10—C11—C12	−0.07 (16)	C25—C26—C27—C28	0.85 (15)
C10—C11—C12—C13	2.07 (16)	C26—C27—C28—C29	1.48 (15)
C11—C12—C13—C14	−0.98 (15)	C27—C28—C29—C30	−1.49 (15)
C12—C13—C14—C9	−2.12 (14)	C28—C29—C30—C25	−0.84 (13)
C12—C13—C14—C15	176.15 (9)	C28—C29—C30—C31	175.30 (9)
C10—C9—C14—C13	4.02 (14)	C26—C25—C30—C29	3.08 (12)
C8—C9—C14—C13	−175.07 (9)	C24—C25—C30—C29	−176.06 (8)
C10—C9—C14—C15	−174.22 (9)	C26—C25—C30—C31	−173.03 (8)
C8—C9—C14—C15	6.69 (14)	C24—C25—C30—C31	7.83 (12)
C2—C1—C15—C14	−119.84 (9)	C29—C30—C31—C17	119.16 (9)
C6—C1—C15—C14	63.99 (11)	C25—C30—C31—C17	−64.75 (10)
C2—C1—C15—C16	111.37 (9)	C29—C30—C31—C32	−112.12 (9)
C6—C1—C15—C16	−64.80 (10)	C25—C30—C31—C32	63.98 (10)
C13—C14—C15—C1	117.00 (9)	C18—C17—C31—C30	−118.43 (9)
C9—C14—C15—C1	−64.75 (11)	C22—C17—C31—C30	63.20 (11)
C13—C14—C15—C16	−113.52 (9)	C18—C17—C31—C32	112.05 (9)
C9—C14—C15—C16	64.73 (11)	C22—C17—C31—C32	−66.32 (10)
C1—C15—C16—O1	157.50 (8)	C30—C31—C32—O2	−157.63 (8)
C14—C15—C16—O1	27.83 (12)	C17—C31—C32—O2	−28.05 (11)
C1—C15—C16—N1	−26.25 (11)	C30—C31—C32—N2	26.99 (11)
C14—C15—C16—N1	−155.92 (8)	C17—C31—C32—N2	156.57 (8)

### Hydrogen-bond geometry (Å, °)

**Table:**

*D*—H···*A*	*D*—H	H···*A*	*D*···*A*	*D*—H···*A*
N1—H1N···O4	0.884 (15)	2.035 (15)	2.9096 (12)	170.2 (13)
O3—H1O···O1	0.927 (18)	1.679 (19)	2.5971 (12)	169.9 (18)
O6—H2O···O2	0.91 (2)	1.66 (2)	2.5517 (12)	168.3 (19)
N2—H3N···O5	0.895 (15)	2.103 (15)	2.9645 (12)	161.2 (14)
N2—H4N···O4	0.843 (16)	2.237 (15)	2.9129 (12)	137.3 (13)
N1—H2N···O5	0.866 (16)	2.151 (16)	2.9088 (12)	145.9 (13)
